# Arginine Methylation in Brain Tumors: Tumor Biology and Therapeutic Strategies

**DOI:** 10.3390/cells10010124

**Published:** 2021-01-11

**Authors:** Jean-Paul Bryant, John Heiss, Yeshavanth Kumar Banasavadi-Siddegowda

**Affiliations:** Surgical Neurology Branch, National Institute of Neurological Disorders and Stroke, National Institutes of Health, Bethesda, MD 20892, USA; jean-paul.bryant@nih.gov (J.-P.B.); heissj@ninds.nih.gov (J.H.)

**Keywords:** arginine methylation, PRMT, cancer, small molecule inhibitors, brain tumor

## Abstract

Protein arginine methylation is a common post-translational modification that plays a pivotal role in cellular regulation. Protein arginine methyltransferases (PRMTs) catalyze the modification of target proteins by adding methyl groups to the guanidino nitrogen atoms of arginine residues. Protein arginine methylation takes part in epigenetic and cellular regulation and has been linked to neurodegenerative diseases, metabolic diseases, and tumor progression. Aberrant expression of PRMTs is associated with the development of brain tumors such as glioblastoma and medulloblastoma. Identifying PRMTs as plausible contributors to tumorigenesis has led to preclinical and clinical investigations of PRMT inhibitors for glioblastoma and medulloblastoma therapy. In this review, we discuss the role of arginine methylation in cancer biology and provide an update on the use of small molecule inhibitors of PRMTs to treat glioblastoma, medulloblastoma, and other cancers.

## 1. Introduction

Arginine methylation is a well-studied post-translational modification that has been implicated as a transcriptional regulator in many cellular processes including cell signaling, DNA damage, and pre-mRNA splicing [1]. Methylation of arginine residues is one of the most common post-translational modifications occurring in mammalian cells. Protein arginine methyltransferases (PRMTs) catalyze this modification of target proteins by adding methyl groups to the guanidino nitrogen atoms of arginine residues. Methylation of arginine residues on proteins modifies protein–protein interactions in various signaling pathways including insulin signaling in glucose metabolism, nongenomic estrogen signaling, nitrous oxide regulation, and activation of numerous downstream proteins [2]. Aberrant expression of proteins responsible for arginine methylation, namely PRMTs, have been implicated in tumorigenesis and other pathological processes [3,4,5,6,7,8,9]. A variety of cancers have been linked to alterations in methyltransferase enzyme activity, including the brain tumors glioblastoma and medulloblastoma. Within the past decade, investigators have increasingly studied the role arginine methylation plays in brain tumors, particularly the relationship of PRMT activity to glioblastoma (GBM) and medulloblastoma development [10,11,12,13,14,15,16].

This review will discuss current literature on the role of arginine methylation in the development of various cancers and the use of arginine methyltransferase inhibitors in cancer therapy, especially glioblastoma and medulloblastoma.

## 2. Functional Significance of Arginine Methylation

Arginine methylation can have both activating and repressive effects depending on the target of the protein methyltransferase [1]. Arginine methylation events that produce activating histone marks are involved in a variety of cellular processes, ranging from cellular differentiation to neural response to stimulatory substances [2,17,18,19]. Mammals produce three main forms of methylarginines: symmetrically dimethylated arginine (SDMA), asymmetrically dimethylated arginine (ADMA), and monomethylated arginine (MMA). The three types of PRMTs are categorized by their catalytic activity [20]. Type I PRMTs include PRMT1, PRMT2, PRMT3, PRMT4/CARM1, PRMT6, and PRMT8, and catalyze the formation of ADMA or MMA [21]. Type II PRMTs consist of PRMT5 and PRMT9 and produce MMA and SDMA [21]. The only type III PRMT is PRMT7, which catalyzes the formation of MMA [22]. It is now widely accepted that these enzymes do not share major redundancy as multiple experiments have delineated distinct functions [23,24,25,26]. Mice knockouts of these enzymes (primarily Type I PRTMs) can lead to a phenotype that is lethal prior to or shortly after birth, underlining the importance of PRMTs in normal physiology [2]. PRMT1, PRMT4/CARM1, PRMT5, PRM7, and PRMT8 have all demonstrated a role in the regulation of neuronal or glial differentiation. The role of certain PRMTs in neural stem cell differentiation, proliferation, and migration likely explains why the overexpression of PRMTs is associated with aggressive tumor biology.

### 2.1. Type I Protein Arginine Methyltransferases

#### 2.1.1. PRMT1

**Figure 1:**
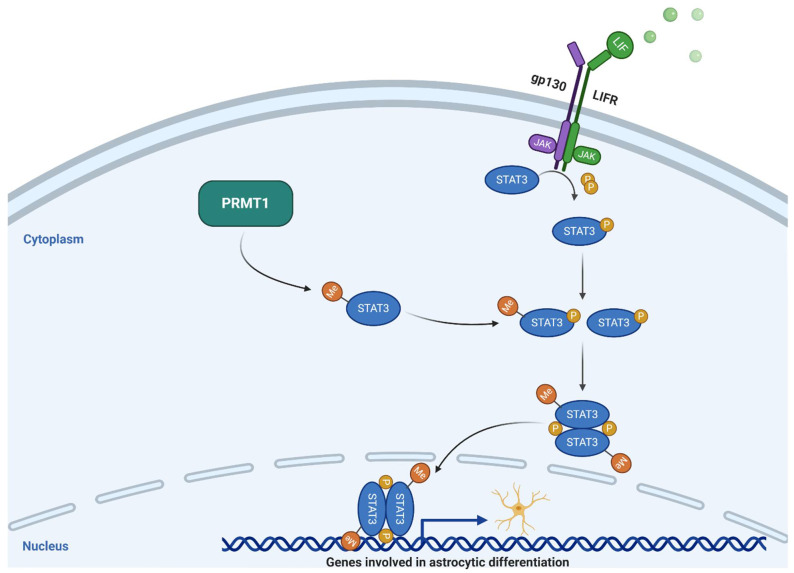
Schematic drawing of the PRMT1-STAT3 axis. JAK = Janus kinase; LIFR = Leukemia inhibitory factor receptor; Me = Methyl; P = Phosphorous; PRMT1 = Protein arginine methyltransferase 1; STAT3 = Signal transducer and activator of transcription 3. Adapted from “PI3K/Akt, RAS/MAPK, JAK/STAT Signaling”, by BioRender.com (2020). Retrieved from https://app.biorender.com/biorender-templates.

PRMT1 primarily catalyzes ADMA, accounting for more than 80% of cellular PRMT activity [21]. This is attributed to its wide substrate specificity with a preference for arginine residues flanked by glycine residues [27]. PRMT1 deposits dimethylarginines on the H4R3 residues and functions as a transcriptional co-activator [2]. PRMT1 interacts with a variety of proteins that influence gene expression and cell-cycle progression including Nuclear Factor Kappa B (NF-ΚB), Sam68, MLL complex, Btg1/Btg2, type I interferon, and hCAF1 [2]. Additionally, PRMT1 has a role in DNA damage signaling and epigenetic regulation of repair pathways that maintain genomic stability [28]. The role of PRMT1 at the cellular level includes the modulation of thermogenic fat activation, maintenance of β-cell identity, renal fibroblast activation, maintenance of normal hematopoiesis, and many other non-neural functions beyond the scope of the present review [29,30,31]. The catalytic activity of PRMT1 has been implicated in numerous neurocellular functions such as the development and monitoring of cells of glial lineage. For example, PRMT1 was found to play an essential role in the central nervous system (CNS) myelination via oligodendrocyte differentiation [32]. In a study conducted by Hashimoto et al., PRMT1 knockout mice displayed significant brain abnormalities with some dying within two weeks of birth [32]. Furthermore, in PRMT1 mutant mice, the number of oligodendrocyte progenitor cells and pre-myelinating oligodendrocytes was significantly reduced, indicating a critical role of PRMT1 in oligodendrocyte differentiation, maturation, and viability [32]. Likewise, PRMT1 has been shown to regulate astrocytic differentiation of neural stem cells in mouse embryos by methylating arginine residues on transcription factor signal transducer and activator of transcription 3 (STAT3) (Figure 1) [33].

In this experiment, knockdown of PRMT1 inhibited promoter activation of glial fibrillary acidic protein (GFAP) in neural stem cells, suggesting its role in positive regulation of neural stem cells [33]. Recently, PRMT1 was shown to play a role in post-translational methylation of SCY1 like pseudokinase 1 (SCYL1), a known regulator of Golgi morphology [34]. SCYL1 interacts with γ_2_COP to form coat protein complex I (COPI) vesicles which regulate Golgi morphology, a process essential for neurite growth. Arginine methylation of SCYL1 facilitates its interaction with γ_2_COP. In this study, inhibition of PRMT1 suppressed both axonal growth and dendritic complexity due to aberrant Golgi morphology [34].

Increased expression of PRMT1 has been implicated in the tumorigenesis of multiple cancers including progesterone receptor positive breast cancer, hepatocellular carcinoma (HCC), neuroblastoma, and pancreatic cancer [35,36,37,38]. PRMT1 is also a known contributor to the development of glioblastoma (GBM) [15,39].

#### 2.1.2. PRMT2

PRMT2 is one of the less characterized PRMTs due to its low activity. However, PRMT2 is known to promote apoptosis via NF-κB dependent mechanism in which NF-κB transcription is inhibited, preventing IκB-α from leaving the nucleus, resulting in increased levels of nuclear IκB-α and decreased NF-κB binding to DNA [40]. PRMT2 is known to interact with a multitude of splicing factors and splicing-related proteins, and other interactions are possible [41]. Hou et al. revealed a role for PRMT2 in dendrite arborization by promoting methylation of the actin nucleator, Cobl [42]. PRMT2 has been implicated in the tumorigenesis of breast cancer through interactions with nuclear hormone receptors and accelerant of hepatocellular carcinoma growth [43,44]. Additionally, enrichment of asymmetric dimethylation of H3R8 (H3R8me2a) is associated with known activating histone marks. This finding has implications in GBM tumorigenesis, in that, PRMT2 has been shown to act as a transcriptional co-activator of genes involved in oncogenesis and more specifically, GBM development [14]. Overexpression of PRMT2 in GBM pathogenesis makes it a potential target for tumor therapy but a potent small molecule inhibitor of PRMT2 has not yet been designed.

#### 2.1.3. PRMT3

The primary function of PRMT3 involves ribosomal protein methylation, which is critical to ribosomal maturation [45,46,47]. Studies describing PRMT3 function in the nervous system are limited to animal models. Mice receiving siRNA oligonucleotides against PRMT3 mRNA showed deformed dendritic spines [46]. PRMT3 localized in normal mice to the dendrites, being absent in cell bodies and axons [46,48]. PRMT3 overexpression has not been associated with brain tumorigenesis but has been implicated in pancreatic cancer and pediatric acute monoblastic leukemia [49,50]. PRMT3 has also been shown to interact with the tumor suppressor DAL-1/4.1B, which inhibits its catalytic activity, promoting apoptosis of breast cancer cells [51].

#### 2.1.4. CARM1/PRMT4

CARM1/PRMT4 functions as a transcriptional coactivator by directly recruiting transcription factors and methylating H3R17 and H3R26 [1]. It also regulates pre-mRNA splicing and mRNA decay through its association with UPF1 [52]. CARM1/PRMT4 itself is regulated by a myriad of microRNAs, including miR-181c, which results in the promotion of human embryonic stem cell differentiation [53]. CARM1/PRMT4 is thought to be involved in hippocampal and motor neuron development and, like PRMT3, hippocampal neurons in PRMT4 deficient rats showed abnormal dendritic spine morphology [54].

**Figure 2:**
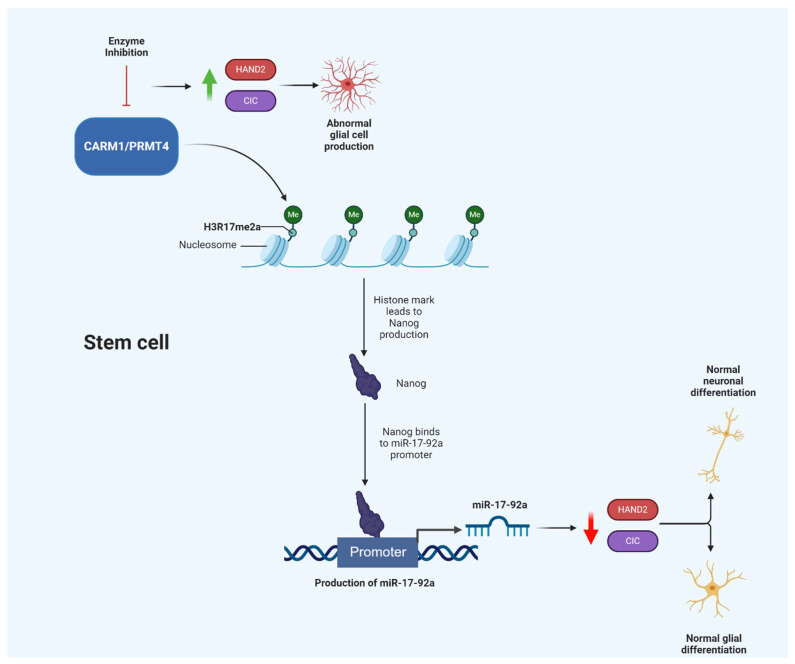
Schematic drawing of the proposed CARM1/PRMT4-miR-17-92a axis. Enzyme inhibition is purported to lead to abnormal glial cell production. CARM1 = Coactivator Associated Arginine Methyltransferase 1; PRMT4 = Protein arginine methyltransferase 4; miR = microRNA. Created with BioRender.com.

The CARM1/PRMT4 relationship with the survival motor neuron (SMN) gene, which, when mutated, promotes spinal muscular atrophy (SMA), has been thoroughly studied [55]. CARM1/PRMT4 was abnormally upregulated in spinal cord tissue from mouse models with SMA. CARM1/PRMT4 was previously shown to interact with the RNA-binding protein HuD and influence SMN expression [56]. Additionally, CARM1/PRMT4 is well-known for producing histone marks that are crucial for astroglial development and differentiation. Inhibition of CARM1/PRMT4, with subsequent loss of the H3R17me2a mark, diminished levels of miR-10a and miR-575 which are purported to be essential entities for astrocyte lineage maintenance [57]. Additionally, CARM1/PRMT4 regulates miR-17-92a production which modulates genes, such as *CIC* and *HAND2*, that influence the differentiation and production of neuronal and glial cells (Figure 2) [57].

CARM1/PRMT4 has been well studied in terms of its association with lung and breast cancer and, more recently, implicated in the development of GBM [58].

#### 2.1.5. PRMT6

PRMT6 is primarily found in the nucleus and possesses specificity for HIV tat, HMGA 1a/b, DNA polymerase beta, and histone H3 [2]. PRMT6 is chiefly responsible for the asymmetric dimethylation of the H3R2 residue producing the H3R2me2a histone mark which is associated with transcriptional repression. PRMT6 is further stabilized by its automethylation properties. The formation of the H3R2me2a histone mark interferes with the generation of the H3Kme3 histone mark by impeding trimethylation of the H3K4 residue [59]. Furthermore, Stein et al. found that PRMT6 and subunits of Polycomb repressor complexes 1 and 2 bind regulatory regions of *HOXA* genes to affect neuronal differentiation [59]. PRMT6 has also been shown to be a potent coactivator of the androgen receptor (AR). PRMT6 can enhance the polyglutamate expanded activity of the AR, implicating its role in spinal bulbar atrophy and SMA by increasing AR toxicity in human motor neurons [60].

#### 2.1.6. PRMT8

PRMT8 is found only in the brain and is localized to the plasma membrane [61]. Increased PRMT8 expression is involved with mouse embryonic stem cells differentiating into neural progenitors [62]. Solari et al. found that the pluripotency associated transcription factors *Oct4*, *Sox2*, and *Nanog*, induced PRMT8 promoter activity in pluripotent stem cells [62]. Furthermore, PRMT8 mRNA levels were increased during neural precursor differentiation [62]. Another study examined the role of PRMT8 as a regulator of retinoid gene expression and cell specification [63]. PRMT8 is a retinoid receptor target gene that is directly expressed in response to the retinoic acid signal and collaborates with PRMT1 to potentiate retinoid response. Knockdown of PRMT1 or PRMT8 eliminated neural specification [63]. Furthermore, PRMT8 depletion produced incoordination in mice by decreasing arborization of cerebellar Purkinje dendrites [64]. This occurred because of the absence of PRMT8 mediated phospholipase activity that normally directly hydrolyzes phosphatidylcholine, an essential step in neurite development. Additionally, PRMT8 depletion increased cellular markers associated with gliomagenesis [63].

### 2.2. Type II Protein Arginine Methyltransferases

#### 2.2.1. PRMT5

Most SDMA in mammalian cells comes from PRMT5 activity. PRMT5 interacts with multiple protein partners through MEP50, which is required for its activation in vitro [65]. PRMT5 heterodimerizes with MEP50 and uses S-adenosyl-L-methionine (SAM) as a cofactor to catalyze the symmetric dimethylation of various histone substrates [66]. Via the same mechanism, PRMT5 catalyzes the methylation of non-histone substrates such as Sm proteins, a process regulated by protein pICln [67]. Another non-histone substrate which is ultimately modified by PRMT5 involves the carboxy terminal domain (CTD) of the RNA polymerase II (RNAP II) subunit POLR2A [68]. The CTD residue R1810 is symmetrically dimethylated yielding the R1810me2s modification, which requires PRMT5 and subsequently is responsible for the recruitment of the Tudor domain of the SMN protein [68]. As previously mentioned, the SMN protein is mutated in SMA and also interacts with senataxin, which can harbor mutations associated with amyotrophic lateral sclerosis [69]. The symmetric dimethylation of RNAP II has also shown to be catalyzed by CARM1 [70].

Neural stem cell proliferation and neural stem cell survival are, in part, epigenetically regulated by the recruitment of PRMT5 to chromatin by Schwann cell factor 1 [71]. PRMT5 modifies histone H4 at arginine 3 through symmetric dimethylation yielding a modification that is correlated with undifferentiated neural stem cells [15,71]. Therefore, PRMT5 regulates the cell’s self-renewal capability and cell-cycle advancement, markedly affecting cellular differentiation and proliferation [71]. In addition to the modification of the H4R3 residue, PRMT5 also catalyzes the symmetric methylation of residues H3R2 and H3R8 producing histone marks H3R2me2s and H3R8me2s [72,73]. Additionally, PRMT5 is essential to neuronal stem cell survival, and depleting it in transgenic mice results in death within 14 days [74]. Given its critical role in regulating neural stem cell proliferation, it is unsurprising that increased expression of PRMT5 has been implicated in tumorigenesis and is associated with worse GBM prognosis [13,15,16,75,76,77].

In addition to modulating the proliferation of neural stem cells, PRMT5 has been implicated in the regulation of oligodendrocyte differentiation. Huang et al. showed that PRMT5 expression was elevated in myelinating oligodendrocytes and that knockdown of PRMT5 inhibited oligodendrocytic cellular differentiation [78]. Furthermore, in a glioma cell line, PRMT5 deficiency induced expression of inhibitors of differentiation (Id) namely, Id2 and Id4 [78]. The study concluded that PRMT5 activity determines the methylation status of CpG islands of both the Id2 and Id4 genes. Ultimately this leads to gene silencing during glial cell proliferation.

#### 2.2.2. PRMT9

The catalytic activity of PRMT9 involves the symmetric dimethylation of spliceosome-associated protein 145 (SAP145) which functions to regulate alternative splicing activity [25,79]. The specificity of PRMT9 and PRMT5 is not redundant as their substrates are distinct. However, the inhibition of PRMT5 in mouse embryos led to almost complete loss of SDMA suggesting that PRMT5 rather than PRMT9 is likely the primary methylator among the type II protein methyltransferases [25]. While literature regarding the pathophysiological contributions made by aberrant expression of PRMT9 is scarce, PRMT9 has been demonstrated to promote hepatocellular carcinoma invasion and metastasis [80].

### 2.3. Type III Protein Arginine Methyltransferases

#### PRMT7

The role of PRMT7 primarily involves transcriptional regulation, generation of snRNPs, and splicing regulation. Additionally, PRMT7 has a role in DNA damage repair because cells depleted of PRMT7 caused de-repression of DNA repair genes and cellular resistance to DNA-damaging agents [81]. This is accomplished by mediating the symmetric dimethylation of H2R3 and H4R3. Furthermore, PRMT7 is involved in stress response and its inhibition significantly reduced levels of Heat Shock Protein 70 (HSP70) stress-associated proteins [82]. Discoveries regarding PRMT7 and the nervous system have been reported recently, with PRMT7 shown to be highly expressed in the hippocampus [83]. Lee et al. have shown that PRMT7 regulates HCN channels in CA1 hippocampal pyramidal cells, which could have implications for neuropsychiatric disorders [83]. Additionally, PRMT7 has been shown to interact with the Beta-catenin-C-Myc axis and promote the growth of renal cell carcinoma (RCC) [84]. The investigators found that overexpressed PRMT7 in RCC cells behaved as an oncogene promoting tumor growth [84]. PRMT7 is currently not associated with brain tumorigenesis. However, in a small series of 10 patients with PRMT7 mutations, one had an orbital tumor, and another had nonspecific brain calcifications, suggesting a role in neuropathogenesis [85].

## 3. Role of PRMTs in Tumorigenesis

### 3.1. Role of Arginine Methylation in Oncogenesis

The expression of PRMTs is generally upregulated in cancer, leading to tumor growth in a variety of organ systems. While many PRMTs have been implicated in cancer development, there are some mechanistic aspects of PRMT activity in oncogenesis that remain unclear. However, the involvement of PRMT5 in metabolic cancer dysregulation has been well studied. For example, Liu et al. investigated the involvement of PRMT5 in promoting lipogenesis and tumor growth [86]. They demonstrated that PRMT5 symmetrically methylates the R321 residue of the sterol regulatory element-binding transcription protein (SREBP), resulting in increased stability. Furthermore, methylation of SREBP by PRMT5 prevented SREBP degradation by promoting evasion from the ubiquitin-proteasome pathway. Subsequently, these events increased lipogenesis and advanced cancer cell growth in both in vivo and in vitro experiments [86]. Since de novo lipogenesis is a vital event in driving the malignant growth of cancer cells, R321 symmetric dimethylation was also associated with clinically advanced disease in patients with HCC and independently predicted a poorer prognosis. In addition to lipogenesis, the ability for cancer cells to utilize aerobic glycolysis for ATP generation is critical to cancer progression. This occurs through reprogramming energy metabolism, where the H^+^-ATP synthase used in oxidative phosphorylation is inhibited by ATPase inhibitory factor 1 (IF1) [87]. The upregulation of IF1 in cancer cells drives a need for a consistent supply and cellular uptake of glucose. In the presence of increased cellular glucose, PRMT5 has been shown to stimulate the release of cyclin dependent kinase 4 (CDK4) from its inhibitor, promoting G1-S transition in cancer cells [88]. Additionally, symmetric methylation of the H3R2 residue in the promoter region of genes involved in gluconeogenesis promotes gene expression of key factors in hepatic glucose production [89]. These studies show the important role PRMT5 plays in the metabolic dysregulation of cancer development.

PRMT5 generates most SDMA in mammals and has been implicated in the pathogenesis of many cancers. PRMT5 expression is increased in leukemia and lymphoma, attributable to its close interaction with myc. Translocations in the myc gene are known to promote Burkitt’s lymphoma [90]. Additionally, PRMT5 activity potentiates hematopoietic stem cell differentiation and hematopoiesis. PRMT5 activity possesses myeloproliferative effects that stem from its interaction with receptor tyrosine kinase FLT3, a protein often mutated in acute myeloid leukemia (AML) [91]. A recent study showed that PRMT5 effects depend on its subcellular location, with PRMT5 nuclear expression associated with prolonged survival and PRMT5 cytoplasmic expression promoting cell growth [92]. This finding has implications for a wide variety of oncologic disease processes, as PRMT5 has been implicated in the pathogenesis of lung adenocarcinoma, lung squamous cell carcinoma, breast cancers, colorectal cancer, and gastric cancer [93,94,95]. Zhang et al. showed that PRMT5 has a role in colorectal cancer pathogenesis through its regulation of arginine methylation of oncogenes eIF4E and FGFR3 [96]. In their experiment, PRMT5 knockdown led to decreased eIF4E and FGFR3 gene expression while PRMT5 was overexpressed in colorectal cancer cells, correlating with decreased overall patient survival. The ability of PRMT5 to act as an oncogene in gastric cancer (GC) cells was described by Kanda et al. [94]. PRMT5 was overexpressed in tumor tissue analysis of 179 patients with GC. Furthermore, PRMT5 knockdown in a GC cell line reduced cellular proliferation and migration. More recently, Liu and colleagues reported that c-Myc directly interacts with PRMT5 and reduced expression of a variety of tumor suppressors [97]. In these experiments, PRMT5 and c-Myc were upregulated in human gastric cancer tissues.

PRMT1 and CARM1/PRMT4 have been implicated in the development of multiple cancers. PRMT1 contributes to the progression of non-small cell lung cancer by methylating the transcription factor Twist1 promoting epithelial-to-mesenchymal transition (EMT) [98]. PRMT1 also regulates EMT in breast cancer cells while CARM1/PRMT4 promotes breast cancer metastasis by methylating arginine residue R1064 of BAF155, a chromatin remodeling factor [1,99]. PRMT7 has also been shown to contribute to breast cancer progression through automethylation, which is believed to play a key role in EMT [100].

Increased PRMT activity is implicated in the development of colorectal cancer. It was recently discovered that type I PRMT inhibition significantly suppressed proliferation and induced apoptosis of colorectal cancer cell lines [101]. PRMT gene expression was also implicated in tumorigenesis in a study examining the L-arginine/NO pathway in esophageal squamous cell carcinoma [102]. In comparison to adjacent healthy tissue, tumors overexpressed *PRMT1*, *PRMT5*, and other genes involved in the L-arginine/NO pathway. Aberrant PRMT5 activity has also been implicated in oral squamous carcinoma (OSCC) where nuclear and cytoplasmic expression of PRMT5 correlated with features of EMT such as loss of E-cadherin and vimentin upregulation [103]. Their findings suggest the possible involvement of PRMT5 in early oncogenesis, progression, and invasion of OSCC.

The effect of arginine methylation on various transcription factors underlines its importance in oncogenesis. It has been reported that PRMT1 associates with MYCN and regulates its stability [104]. Depletion of PRMT1 in this report reduced the expression of MYCN and cell viability in primary neuroblastoma tumors [104]. Overexpression of MYCN is associated with poor outcomes in patients with neuroblastoma and correlates with a high-risk phenotype [105]. In addition to MYCN, NF-KB is a transcription factor that plays an important role in oncogenesis. Reintjes et al. found that PRMT1 forms a complex with NF-KB via interaction with its RelA domain and asymmetrically methylates its R30 residue [106]. This modification can interfere with NF-KB-DNA binding thus affecting the ability of NF-KB to amplify cancer related genes. Importantly, PRMT5 has also been implicated in the regulation of p53 response [107]. It was found that a stress response protein, Strap, recruits PRMT5 to p53 upon DNA damage, facilitating the symmetric methylation of p53 [107]. PRMT5 was also found to influence the functional outcome of p53 response, suggesting an important role for PRMT5 and the ability of p53 to function as a tumor suppressor.

### 3.2. Role of Arginine Methylation in Brain Tumor Development

#### 3.2.1. Glioma

The notion that PRMT1 is involved in the regulation of glial cells was supported by Wang et al. who examined the role of PRMT1 in gliomagenesis (Table 1) [39]. In their experiment, immunohistochemical staining of grade II gliomas showed a strong positive signal for PRMT1 as compared to a weak signal in normal brain tissue, with more than 76% of glioma samples having increased PRMT1 expression. Moreover, PRMT1 knockdown in glioma cells significantly reduced the cell population in the S phase and significantly decreased proliferation rates. In vivo, PRMT1 knockdown significantly reduced tumor growth [39]. From a mechanistic standpoint, these study results suggest a possible role for PRMT1 induction in post-mitotic cellular processes. This was evident by an association of PRMT1 knockdown with suppression of glioma proliferation through cell cycle arrest in G1-S. This finding combined with an increased number of apoptotic glioma cells following PRMT1 depletion suggests a role for PRMT1 not only in cell cycle progression but also in glioma cell apoptosis [39].

While there are few studies investigating the role of PRMT1 in gliomagenesis, Zheng et al. recently published results delineating the role of cancer promoting, long noncoding RNA NNT-AS1 and its interaction with the miR-494-3p-PRMT1 axis (Table 1) [110]. They found that NNT-AS1 inhibition by siRNA diminished glioma cell proliferation, migration, and overall cell viability. Furthermore, miR-494-3p overexpression and PRMT1 inhibition attenuated both glioma cell proliferation and metastasis [110].

The role of PRMT5 is not only critical to glial cell differentiation but also is important in human glioma progression [106,107,110]. In a study examining the role of long noncoding RNA (LINC00515) that exhibits increased expression in human gliomas, Wu et al. found that LINC00515 activated PRMT5 expression, promoted cell growth, and inhibited apoptosis of glioma cells [112]. Another investigation supported the role of long non-coding RNA in gliomagenesis (Table 1) [108]. LncRNA SNHG16 was highly expressed in glioma cell tissues and associated with poorer clinical prognosis [108]. Results indicated that SNHG16 can perform as an oncogene by sponging miR-4518 leading to the upregulation of PRMT5 expression in glioma [108]. This experiment revealed a novel SNHG16-miR-4518-PRMT5 pathway and further supports a role for PRMT5 in tumorigenesis. In addition to the propagation of glial cell lineage, PRMT5 is hypothesized to be involved in tumor angiogenesis. The cardinal study describing this phenomenon found that homeobox C10 (HOXC10) overexpression increased the ability of glioma cells to migrate and proliferate as well as increasing neovascularization by binding to the promoter of vascular endothelial growth factor A (VEGFA) and increasing its expression (Table 1) [109]. Interestingly, PRMT5 was required for the overexpression of VEGFA mediated by HOXC10 representing a potential target for anti-VEGF therapy in glioma therapeutics.

#### 3.2.2. Glioblastoma

**Figure 3:**
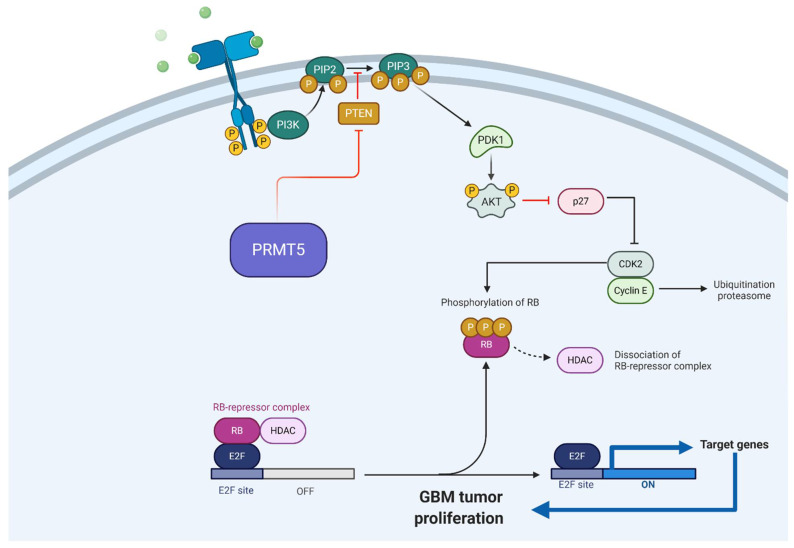
Schematic drawing of PRMT5-PTEN axis. Akt = Protein kinase B; CDK2 = Cyclin-dependent kinase 2; GBM = Glioblastoma; HDAC = Histone deacetylase; PIP2 = Phosphatidylinositol (4,5)-bisphosphate; PIP3 = Phosphatidylinositol (3,4,5)-triphosphate; P = Phosphorous; PTEN = Phosphatase and tensin homolog; PI3K = Phosphoinositide-3-kinase; PRMT5 = Protein arginine methyltransferase 5; RB = Retinoblastoma. Adapted from “G1/S Checkpoint”, by BioRender.com (2020). Retrieved from https://app.biorender.com/biorender-templates.

Among subcategories of brain tumors, GBM is the most well studied regarding its association with arginine methylation [12,13,16,75,113,114,115]. This related to the importance of GBM as the most common and deadly malignant brain neoplasm [116]. The mainstays of GBM treatment are maximal surgical resection followed by radiation and administration of temozolomide or other alkylating chemotherapeutic. Despite this regimen, the 5-year survival rate is dismal and there is a pressing need to develop more effective treatments, including those with new molecular targets. Among the protein arginine methyltransferases implicated in GBM progression, PRMT5 has emerged as the most suitable target [75]. Investigators, including our group, have demonstrated that an increase in PRMT5 expression is associated with a worse GBM prognosis; furthermore, studies have reported a direct correlation between PRMT5 expression and grade of glioma malignancy (Table 1) [76,77]. A study by Han et al. found PRMT5 expression predominate in the nucleus of all GBM tumor specimens (Table 1) [76]. Moreover, epigenetic regulation by Myc and its inhibitor Omomyc have been shown to be associated with PRMT5 expression and influence GBM tumorigenesis [12]. Inhibition of PRMT5 rescued Myc inhibition by Omomyc, suggesting a possible role of PRMT5 in *MYC* target gene silencing. The consistent association found between Myc or Omomyc and PRMT5 suggests that Omomyc may interfere with Myc-PRMT5 interactions and therefore explain a potential role for Omomyc in its established anti-oncogenic properties (Table 1) [12]. Furthermore, Favia et al. demonstrated that PRMT1 and PRMT5 are responsible for the asymmetric and symmetric (respectively) dimethylation of Myc in GBM cells, which regulates the protein’s stability [15]. The alteration in stability affected the biological properties of GBM stem cells [15]. Our group investigated and identified the PRMT5-PTEN pathway for its association with GBM development and proliferation (Figure 3) (Table 1) [13].

We further examined the role of PRMT5 concerning stemness and differentiation status of the patient-derived primary GBM cells (Figure 3); PRMT5 regulates the proliferation and self-renewal of GBM stem-like cells, while it is required for the survival of differentiated GBM cells [13]. Additionally, it was found that PRMT5 positively regulates AKT and ERK activity, while PRMT5 depletion led to increased transcript and protein expression of PTEN in GBM stem-like cells [13]. PRMT5 has also been demonstrated to play a role in GBM therapeutic resistance, highlighting an elusive mechanistic aspect that contributes to the difficulty in long term management of this pathology. This was described in an experiment by Holmes et al. who investigated the role of PRMT5 in therapy resistance (Table 1) [16]. Inhibition of mTOR stimulated PRMT5 activity whereas PRMT5 knockdown sensitized GBM cells to therapeutic agents [16]. Taken together, these results indicate an important role for arginine methylation in glioblastoma tumor biology, particularly driven by the overexpression of PRMT5.

#### 3.2.3. Medulloblastoma

Medulloblastoma is the only brain tumor other than GBM that has been associated with increased PRMT activity. Medulloblastoma is the most common type of malignant pediatric brain tumor, comprising 20% of all childhood brain cancers [117]. Medulloblastomas are classified by their molecular subgroups including Sonic Hedgehog (SHH), wingless (WNT), Group 3, and Group 4 [118]. Group 3 (more specifically group 3γ) medulloblastomas carry the worst prognosis, with less than 50% survival, and are associated with *MYC* amplification [118]. Group 3 medulloblastoma is also associated with aberrant expression of PRMT5. PRMT5 expression correlates with *MYC* expression in both primary medulloblastoma and medulloblastoma cell lines [111]. This was shown by Chaturvedi et al. who investigated PRMT5-Myc interaction in myc-driven medulloblastoma cells to determine the functional role of PRMT5 in medulloblastoma [111]. In 491 medulloblastoma samples, PRMT5 expression was found to be significantly overexpressed on mRNA analysis compared to 9 normal cerebellar controls. Additionally, knockdown of PRMT5 led to a reduction in *MYC* expression as well as a statistically significant reduction in cell growth in medulloblastoma cell lines. Lastly, co-immunoprecipitation from cell extracts showed the presence of PRMT5 in myc-immunoprecipitated complexes in medulloblastoma cells [111]. This study is the only of its kind to demonstrate an association between PRMT5 and *MYC* in medulloblastoma.

## 4. PRMT Inhibition and Clinical Applications

In 2004, Cheng et al. conducted a landmark study identifying small molecules capable of regulating PRMT activity [119]. Their experiments led to the discovery of a primary compound that specifically inhibits arginine methylation, AMI-1. AMI-1 and other AMI compounds were the first small molecule PRMT inhibitors reported to have activity against PRMT1, PRMT3, PRMT4, and PRMT6. Because PRMT5 is the predominant type II arginine methyltransferase, the inactivity of the AMI series against this enzyme limits their effect. However, since then, numerous small molecule inhibitors have been developed and validated assays as PRMT5 and PRMT7-specific inhibitors [120]. In this section, we review the inhibitors specific to each protein arginine methyltransferase.

### 4.1. Type I Protein Arginine Methyltransferases Inhibitors

#### 4.1.1. PRMT1

Because PRMT1 is the predominant enzyme responsible for asymmetric dimethylation and has shown robust recombinant enzyme activity in bacteria, it is the most frequently used enzyme for inhibition testing. While the AMI compounds were among the first general PRMT inhibitors tested, inhibitors such as Allantodapsone, E84, and DB75 also possessed PRMT1 specific inhibitory activity (Table 2). DB75 was approved for clinical use in the U.S. as an antiparasitic treatment and later found to be highly specific PRMT1 inhibitor [120]. Carbocyanine and diamidine compounds have also demonstrated high specificity over other PRMT enzymes with high potency [121]. Some of these inhibitors were efficacious in pre-clinical testing against breast cancer cell lines (Allantodapsone) and various types of leukemia (E84 and DB75) [122]. Moreover, molecular modeling led to the discovery of a PRMT1 inhibitor that significantly inhibits the cancer cell lines, HepG2 [123]. PRMT1 inhibitors can target the Asp84 binding site on PRMT1, a finding that can spur the development of other selective pharmaceutical agents against PRMT1. Most recently, in vivo studies by Zheng et al. found that a combination of PD-L1 checkpoint inhibition and a novel PRMT1 inhibitor (PT1001B) upregulated tumor-infiltrating CD8 T-cells in pancreatic tumors [124]. Furthermore, the inhibitor augmented the anti-tumor effects of anti-PD-L1 antibodies on tumor cell progression while increasing tumor apoptosis. This is one of few studies combining arginine methylation inhibition with immunotherapy, which is likely an area for further investigation.

Currently, there is one clinical trial being conducted which is examining the effect of a PRMT1 inhibitor on Diffuse Large B-cell Lymphoma (Drug: GSK3368715; NCT03666988). Results have yet to be reported for this trial.

#### 4.1.2. PRMT3

The first described inhibitor of PRMT3, compound 1, exhibited allosteric binding and showed promise as an emerging therapy [129]. SGC707 was an analog that demonstrated improved selectivity and potency over the parent compound in pre-clinical testing of adenocarcinoma (Table 2) [45,130].

#### 4.1.3. PRMT4/CARM1

The structure of the first inhibitors of PRMT4/CARM1 described in 2011 and, in 2016 were modified to create a more potent and selective inhibitor, SGC2085 (Table 2) [131,132]. TP-064 was subsequently developed with improved potency but differed in that it was the first cell-active chemical probe for PRMT4/CARM1 [122]. Pre-clinical testing for PRMT4/CARM1 has included the use of another inhibitor, TBBD, in non-small cell lung carcinoma cell lines. Guo et al. also modified the structure of a previously discovered weak dual PRMT4/PRMT6 inhibitor to develop a novel and highly selective PRMT4 inhibitor (compound 49), which induced cell cycle arrest and apoptosis in AML cell lines [126].

#### 4.1.4. PRMT6

High potency inhibitors of PRMT6 were discovered through the modification of the structure of type I PRMT inhibitors. These inhibitors displayed a ~12-fold or greater specificity for PRMT6 compared to other PRMTs. The first identified inhibitor of PRMT6, named EPZ020411, was a novel aryl pyrazole that showed good bioavailability in the in vivo studies (Table 2) [133]. EPZ020411 proved to be 100-fold selective for PRMT6, PRMT8, and PRMT1 compared to PRMT3, -4, and -5. PRMT6 overexpression has been linked to the development of melanoma [122] and breast cancer cell lines have undergone preclinical testing of PRMT6 inhibitors. An analog of the PRMT4/CARM1 inhibitor 6′-methyleneamine sinefungin (GMS) was discovered in 2016 by Wu et al. (Table 2) [134]. GMS inhibited PRMT6 activity significantly better than other cofactor-competitive inhibitors. Subsequently, a potent PRMT6-specific inhibitor MS023 was developed and served as a framework to create the dual PRMT4/CARM1 and PRMT6 inhibitor, MS049 (Table 2) [20,135].

### 4.2. Type II Protein Arginine Methyltransferases

#### PRMT5

PRMT5 is a major type II protein arginine methyltransferase and overexpressed in numerous malignancies, making it an attractive therapeutic target. For example, PRMT5 is a potential target for the inhibition of prostate cancer growth as it potentiates prostate cancer tumorigenesis by methylating AR genes important for tumor progression [136]. The first PRMT5-specific inhibitor, compound 13 (CMP5), and the second, EPZ015666, was discovered in 2015 (Table 2) [127,137]. EPZ015666 was much more specific to PRMT5 than other PRMTs and more potent [122]. EPZ015666 showed significant antitumor effects in mice implanted with Z-138 and Maver-1 xenografts and significantly decreased the symmetric methylation level of these tumors in a dose-dependent fashion [120]. Since the efficacy of EPZ015666 was established, it has been used in multiple experiments to potently inhibit PRMT5 in animal models of glioblastoma and medulloblastoma tumorigenesis [15,111]. Two studies reported that EPZ015666 significantly suppressed cell growth and induced apoptosis suggesting a role for this small molecule inhibitor in glioblastoma and medulloblastoma therapy [15,111]. Previously, our group showed that CMP5 is efficacious in inhibiting PRMT5 activity in an in vitro GBM model as well as an in vivo glioma zebrafish model [75]. Among the four agents tested in our study, CMP5 exhibited consistent specificity for PRMT5, blocked cell-cycle progression in GBM neurospheres, and drove GBM neurospheres toward the senescent phenotype [75]. Our study was one of the first investigations to show that PRMT5 is a druggable target for GBM therapy. More recently, Zhu et al. evaluated 44 compounds for their inhibitory effects on PRMT5 in vitro [138]. Their top two active inhibitors induced cell cycle arrest and apoptosis in MV4-11 cells [138]. Similarly, Bonday et al. identified a potent PRMT5-specific inhibitor, LLY-283, which displayed antitumor activity in mouse xenografts [139]. Five clinical trials examining the use of PRMT5-specific inhibitors on various malignancies are currently recruiting patients. These trials include two multicenter, open-label, dose-escalation studies examining the effect of PRMT5 inhibition on brain tumors (Table 2). One of these multicenter studies (NCT04089449) is evaluating the efficacy of the inhibitor PRT811 on advanced solid tumors and GBM while the other multicenter trial (NCT02783300) is evaluating inhibitor GSK3326595 on solid tumors, including GBM and Non-Hodgkin’s Lymphoma NHL. The remaining trials are evaluating the safety and efficacy of PRMT5 inhibition are in patients with advanced/metastatic solid tumors (Drug: PF-06939999; NCT03854227), Myelodysplastic Syndrome (MDS) and AML (Drug: GSK3326595; NCT03614728), and NHL/MDS (Drug: JNJ64619178; NCT03573310).

### 4.3. Type III Protein Arginine Methyltransferases

#### PRMT7

Smil et al. designed the first inhibitor of PRMT7 which had an IC_50_ value of 6 µM against PRMT7 and PRMT5 [140]. The dual inhibitor, named DS-437, was inactive against 29 human methyltransferases and underwent pre-clinical testing against breast cancer cells. Recently, a potent and cell-active chemical probe for PRMT7 was discovered [82]. This PRMT7-specific inhibitor, named SGC3027, inhibits in vitro methylation of HSP70 at the R469 residue (Table 2). This modification of a cellular stress-response protein is typically driven by PRMT7 and inhibition of this process decreases the cell’s ability to adapt to disruptions in proteostasis. The discovery of this inhibitor added to the increasing therapeutic options for PRMT inhibition and further outlined the role of PRMT7 in the stress response [82].

### 4.4. Future Directions for Arginine Methylation Inhibition in Brain Tumors

PRMT inhibition has emerged as a viable therapeutic strategy in numerous malignancies. In brain cancer, PRMT inhibition is garnering more attention as these enzymes, specifically PRMT5, are targeted in clinical trials. PRMT5 was the first methyltransferase to be targeted in clinical trials because its overexpression and role in GBM pathogenesis was documented more extensively than the other PRMTs. PRMT5 inhibition is also being tested for medulloblastoma therapy. The Chaturvedi et al. clinical study of patients with the most aggressive subtype of medulloblastoma used EPZ015666 to inhibit PRMT5 [111]. Further study is needed to evaluate the efficacy of EPZ015666 in inhibiting medulloblastoma growth and the role of PRMT5 in medulloblastoma tumorigenesis. Inhibitors of PRMTs other than PRMT5 will be in clinical trials if they demonstrate potent antitumor effects in preclinical testing. A promising target is PRMT1 because it is overexpressed in glioblastoma. Existing PRMT1 inhibitors, E84 and DB75, that are currently used for non-solid tumors could be tested against GBM.

## 5. Conclusions

The role of aberrant PRMT expression in the pathogenesis of many malignancies has garnered increased attention recently. The association between PRMT5 and GBM has led to multiple in vitro and in vivo studies regarding the role of this enzyme in GBM pathogenesis and its inhibition in GBM treatment. Additionally, other PRMTs have been implicated in brain cancer pathogenesis and neural stem cell dysregulation suggesting their suitability as future therapeutic targets for GBM. In addition to tumors affecting the nervous system, aberrant PRMT expression has been associated with a variety of solid tumors such as colorectal cancer and ovarian cancer (among others) as well as hematologic cancers including AML and NHL. Links between PRMT expression and tumorigenesis continue to be discovered and some experimental pharmaceutical therapies with small molecule PRMT inhibitors have completed pre-clinical testing and are now being tested in clinical trials. PRMTs represent a druggable target for therapies that could improve the treatment of malignant brain tumors such as glioblastoma and medulloblastoma. Furthermore, PRMTs may be suitable therapeutic targets for other solid cancers and hematologic malignancies.

## Figures and Tables

**Table 1:** Summary of studies associating PRMT activity with brain tumorigenesis. GBM = Glioblastoma; VEGFA = Vascular endothelial growth factor-A; LncRNA = Long non-coding RNA.

**Brain Tumor Type**	**Investigators** **(Year)**	**Enzyme**	**Study Conclusions**
**Glioma**	Wang et al. [39] (2012)	PRMT1	PRMT1 was upregulated in glioma tissues compared to normal cortex tissue. PRMT1 knockdown resulted in G1-S arrest in four glioma cell lines. RNAi greatly reduced tumor growth in vivo.
	Lu et al. [108] (2018)	PRMT5	LncRNA SNHG16 knockdown inhibited glioma cell proliferation and induced apoptosis. SNHG16 up-regulated expression miR-4518 targeted gene PRMT5 via sponging of miR-4518.
	Tan et al. [109] (2018)	PRMT5	PRMT5 was required for HOXC10-mediated upregulation of VEGFA. HOXC10 levels and VEGFA expression correlated significantly in human glioma.
	Zheng et al. [110] (2020)	PRMT1	LncRNA NNT-AS1 is significantly up-regulated during the early stages of glioma in vitro. Inhibition of NNT-AS1 led to positive regulation of PRMT1 via miRNA-494-3p.
**Glioblastoma**	Yan et al. [77] (2014)	PRMT5	PRMT5 attenuation limited recruitment to the promoter of tumor suppressor ST7. Chromatin immunoprecipitation and genetic profiling showed that the ST7 gene is silenced by PRMT5. PRMT5 overexpression in primary GBM and cell lines correlated positively with cell growth and inversely with overall survival.
	Han et al. [76] (2014)	PRMT5	Protein expression profiles revealed that PRMT5 expression was low in low grade glial cell controls and low grade astrocytomas. PRMT5 expression was high in GBM and increased in parallel with malignant progression.
	Mongiardi et al. [12] (2015)	PRMT5	Myc and Omomyc stimulated PRMT5-mediated symmetric dimethylation of H4R3 in human GBM cells. Myc and Omomyc are consistently associated with PRMT5. PRMT5 interference impaired gene activation by Myc.
	Banasavadi-Siddegowda et al. [13] (2017)	PRMT5	PRMT5 depletion caused senescence and apoptosis in the patient-derived primary stem-like cells and differentiated cells respectively. PRMT5 depletion stunted the tumor growth and increased the survival of mice in the intracranial GBM tumor model.
	Holmes et al. [16] (2019)	PRMT5	PRMT5 inhibition by EPZ015666 and PP42 displayed synergistic effects in vitro and a mouse model.
**Medulloblastoma**	Chaturvedi et al. [111] (2019)	PRMT5	PRMT5 knockdown significantly decreased medulloblastoma cell growth. PRMT5 inhibition with EPZ015666 suppressed cell growth and induced apoptosis of myc-driven medulloblastoma cells in a dose-dependent manner.

**Table 2:** Small molecule inhibitors of PRMT enzymes. CML = Chronic myeloid leukemia; NSCLC = Non-small cell lung carcinoma; GBM = Glioblastoma; MCL = Mantle cell lymphoma; DLBCL = Diffuse large B-cell lymphoma; NHL = Non-Hodgkin lymphoma; MM = Multiple myeloma; AML = Acute myeloid leukemia; APL = Acute promyelocytic leukemia.

**Enzyme**	**Enzyme Target(s)**	**Inhibitor(s)**	**Pre-Clinical Testing [REF]**	**Clinical Trial**
**PRMT1**	H4R3, H2AR3	Allantodapsone	Breast cancer [125]	
		E84	CML, AML	
		DB75	CML, AML, APL [121]	
		PT1001B	Pancreatic cancer [124]	
		GSK3368715		NCT03666988
**PRMT3**	Ribosomal protein-RPS2	7 SGC707		
**PRMT4/CARM1**	H3R2, H3R17, H3R26 RNAP II	CMPD-1 CMPD-2 Compound 49		
		SGC2085 TP-064	AML [126]	
		TBBD		
**PRMT5**	H3R2,H3R8, H4R3, H2AR3 Sm proteins Nuclear/cytoplasmic proteins RNAP II	CMP5	GBM [75]	
		EPZ015666	NHL, MCL, GBM, Breast cancer, MM [127]	
		GSK3326595	AML/MDS [128]	NCT02783300, NCT03614728
		JNJ64619178		NCT03573310
		LLY-283		
		PF-06939999		NCT03854227
		PRT811		NCT04089449
**PRMT6**	H3R2, H2AR9, H4R3	EPZ020411 6′-methyleneamine sinefungin (GMS) MS023 MS049		
**PRMT7**	H4R3, H2AR3, H3R2	DS-437 SGC3027		

## Data Availability

Not applicable.
